# Ammonia Toxicity and Associated Protein Oxidation: A Single-Cell Surface Enhanced Raman Spectroscopy Study

**DOI:** 10.1021/acs.chemrestox.3c00368

**Published:** 2023-12-26

**Authors:** Davide Redolfi-Bristol, Alessandro Mangiameli, Kenta Yamamoto, Elia Marin, Wenliang Zhu, Osam Mazda, Pietro Riello, Giuseppe Pezzotti

**Affiliations:** † Ceramic Physics Laboratory, Kyoto Institute of Technology, Sakyo-ku, Matsugasaki, Kyoto 606-8585, Japan; ‡ Department of Molecular Genetics, Institute of Biomedical Science, Kansai Medical University, 2-5-1 Shinmachi, Hiraka-ta, Osaka 573-1010, Japan; § Department of Immunology, Graduate School of Medical Science, Kyoto Prefectural University of Medicine, Kamigyo-ku, 465 Kajii-cho, Kyoto 602-8566, Japan; ∥ Department of Dental Medicine, Graduate School of Medical Science, Kyoto Prefectural University of Medicine, Kamigyo-ku, Kyoto 602-8566, Japan; ⊥ Department of Orthopedic Surgery, Tokyo Medical University, 6-7-1 Nishi-Shinjuku, Shinjuku-ku, Tokyo 160-0023, Japan; # Department of Applied Science and Technology, Politecnico di Torino, Corso Duca degli Abruzzi 24, Torino 10129, Italy; ¶ Dipartimento di Scienze Molecolari e Nanosistemi, Università Ca’ Foscari di Venezia, Via Torino 155, Venezia 30172, Italia

## Abstract

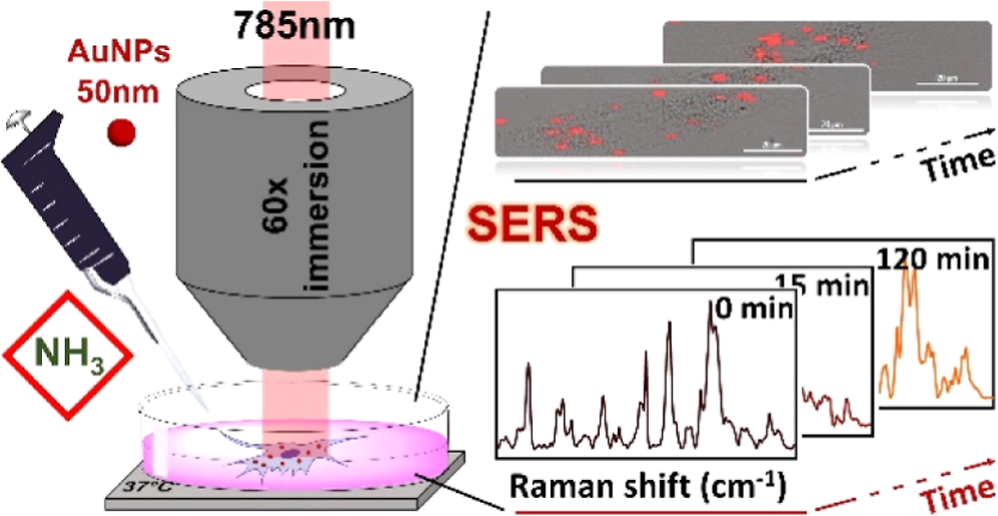

Ammonia (NH_3_) is a commonly used industrial chemical to which exposure at high concentrations can result in severe skin damage. Moreover, high levels of ammonia in the human body can lead to hyperammonemia conditions and enhanced cancer metabolism. In this work, the toxicity mechanism of NH_3_ has been studied against human dermal fibroblast (HDF) cells using surface-enhanced Raman spectroscopy (SERS). For this purpose, gold nanoparticles of size 50 nm have been prepared and used as probes for Raman signal enhancement, after being internalized inside HDF cells. Following the exposure to ammonia, HDF cells showed a significant variation in the protein ternary structure’s signals, demonstrating their denaturation and oxidation process, together with early signs of apoptosis. Meaningful changes were observed especially in the Raman vibrations of sulfur-containing amino acids (cysteine and methionine) together with aromatic residues. Fluorescence microscopy revealed the formation of reactive oxygen and nitrogen species in cells, which confirmed their stressed condition and to whom the causes of protein degradation can be attributed. These findings can provide new insights into the mechanism of ammonia toxicity and protein oxidation at a single-cell level, demonstrating the high potential of the SERS technique in investigating the cellular response to toxic compounds.

## Introduction

Ammonia (NH_3_) is a highly toxic substance that can cause severe damage to living cells when they are exposed to high concentrations.^1−3^ After ammonia exposure, cells can experience a wide range of detrimental effects, including activation of inflammatory response, protein denaturation, oxidative stress, and apoptosis.^4−7^ Indeed, NH_3_ is known to disrupt cellular homeostasis by altering intracellular pH, inducing mitochondrial dysfunction, and increasing the production of reactive oxygen species and reactive nitrogen species (ROS and RNS, respectively).^6−8^ Prolonged exposure to high levels of ammonia can result in a variety of health issues such as respiratory distress, central nervous system damage, liver damage, and even death. Ammonia toxicity is generally associated with occupational hazards, such as exposure to industrial chemicals, and can have serious implications for human health and the environment.^1,3^

In addition to ammonia exposure caused by external factors, high levels of ammonia in the human body can be found in patients with acute or chronic liver diseases.^9,10^ NH_3_ is a toxic waste product formed in the body during the digestion of protein. Under normal circumstances, ammonia is processed in the liver, where it is converted into urea and eliminated through the urine.^11,12^ However, if ammonia is not correctly processed, it can accumulate in the bloodstream and lead to the hyperammonemia condition, whose symptoms include nausea, vomiting, headache, decreased muscle tone, and neurodevelopmental delays.^6,9,13−15^ Eventually, it has recently been demonstrated that cancer cells are able to recycle waste ammonia into core amino acid metabolism, maximizing nitrogen utilization and thus accelerating tumor proliferation.^16,17^

Gaining a precise understanding of the various physiochemical alterations that occur as a result of exposure to high concentrations of ammonia is therefore crucial to improving our understanding of cellular responses and consequently lead to the development of more effective therapeutics. However, the biomolecular events triggered by ammonia and ROS/RNS are highly dynamic, which makes it challenging to simultaneously observe the chemical and conformational changes in the involved biomolecules. To overcome this hurdle, precise and sensitive methods are required to enable the comprehensive visualization of these complex biomolecular events.

Raman spectroscopy is a technique that enables direct assessments of chemical bonds through their specific vibrational signatures.^18,19^ In recent years, many biological uses of Raman methods have been developed, such as the observation of cellular metabolism and physiology,^20,21^ disease identification,^22,23^ and cell differentiation.^24,25^ This is due to its nonintrusive nature and the possibility to distinguish between the spectral signatures of many different biomolecules, which makes it optimal for biomedical applications.^26,27^ However, the use of simple Raman microspectroscopy to study biological samples can have different disadvantages depending on the measurement condition, such as Raman scattering efficiency, sample autofluorescence, and photodamage. Indeed, to obtain sufficient Raman scattered signals and reduce the acquisition times, short-wavelength lasers are preferentially used. These lasers, however, can cause larger autofluorescence from the sample and a more pronounced photodamaging phenomenon, thus affecting the results. The use of a near-infrared laser can decrease the photodamage to biological samples (since they are transparent to it); however, it will requires longer acquisition times to increase the Raman scattering efficiency.^28^ These combined effects impair the possibility to perform in-time studies and to analyze rapid biomolecular changes. To overcome these drawbacks and further increase the potential of the Raman microspectroscopy technique, surface enhanced Raman spectroscopy (SERS) has been extensively investigated and applied. SERS is a method in which inelastic light scattering deriving from molecules adsorbed onto metal surfaces and or/nanoparticles is greatly enhanced due to the resonant light–metal–molecule interactions.^29,30^ This phenomenon can be exploited to detect smaller variations in biological events with higher Raman scattering efficiency and reduced times.^31,32^ Additionally, the use of nanoparticles as SERS probes allows an accurate analysis of the metabolic processes that occur at a single-cell level, thanks to their possible internalization inside the cell.^33,34^

In this study, we used gold nanoparticles (AuNPs) of size 50 nm as SERS probes to investigate the effects of NH_3_ exposure on cells, using human dermal fibroblast (HDF) cells as the model. The SERS technique allowed the detection of protein oxidation and denaturation over time, caused by the formation of ROS and RNS inside the cell. Sulfur-containing amino acids (cysteine and methionine) were observed to convert into their oxidized species (cysteic acid and methionine sulfoxide), while phenylalanine and tryptophan residues showed increased interactions with AuNPs during protein denaturation. Fluorescence microscopy was used to visualize and confirm ROS/RNS generation in cells when they were continuously exposed to ammonia. Our work shows the advanced application of SERS in real-time monitoring of dynamic and complex biomolecular changes in single-living cells, revealing new details on the toxic mechanism of ammonia and the alterations in the protein structure caused by the formation of ROS and RNS.

## Material and Methods

### Materials

Tetrachloroauric acid (HAuCl_4_·3H_2_O), sodium citrate tribasic dihydrate, and 2′,7′-dichlorofluorescein diacetate (DCFH-DA) were purchased from Sigma-Aldrich. Dulbecco’s modified Eagles’ medium (DMEM), fetal bovine serum (FBS), MEM nonessential amino acid solution, l-sodium pyruvate, penicillin–streptomycin mixed solution, dl-methionine, and phosphate-buffered saline (PBS) solutions were purchased from Nacalai Tesque. Calcein-AM and propidium iodide (PI) solutions were purchased from Dojindo. 10% ammonia solution, *tert*-butyl hydroperoxide (tBHP), l-cysteine, l-cysteic acid, and dl-methionine sulfoxide were acquired from FUJIFILM Wako Pure Chemical Corporation.

### Synthesis of AuNPs

AuNPs of size 50 nm have been prepared following a modified method from the one reported by Bastus et al.^35^

#### Synthesis of the AuNPs Seed of Size 30 nm

In a two-neck round-bottom flask equipped with a bubble condenser was brought to boil 23.8 mL of HAuCl_4_·3H_2_O (0.5 mM) in an oil bath under vigorous stirring. When the solution was boiling, 0.7 mL of sodium citrate (34 mM) was injected in the flask. The solution was left to stir for 15 min, allowed to cool down to room temperature, and finally stored at 4 °C. The final seed solution concentration was around 4.1 × 10^11^ NPs/mL, calculated from the initial concentration of gold *C*_Au_ (mol/L)^36^
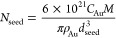
where ρ is the density of gold (19.3 g/cm^3^), *M* is its atomic weight (197 g/mol), and *d* is the average NP diameter obtained from the analytical centrifuge.

#### Synthesis of AuNPs of Size 50 nm

In a two-neck round-bottom flask equipped with a bubble condenser were added 7 mL of seed solution, 3 mL of Milli-Q water, and 1 mL of sodium citrate (15 mM). The solution was placed in an oil bath at 90 °C under stirring, and when the temperature was steady, 1 mL of HAuCl_4_ (2.5 mM) was injected into the vessel. After 30 min, another milliliter of HAuCl_4_ (2.5 mM) was added to the solution. After another 30 min, the first growth step was completed. The resulting solution was used as a new seed solution, and the process was repeated. The obtained colloid was stored at 4 °C, and the final concentration of AuNPs was around 8.5 × 10^10^ NPs/ml, calculated from the number of particles in the volume of the seed solution used.

### Characterization of AuNPs

#### UV–Vis Spectroscopy

UV–vis spectra were acquired with a UV–vis Cary 100 Spectrophotometer (Agilent). AuNPs solution was placed in a glass cuvette, and spectral analysis was performed in the 400–800 nm range at room temperature.

#### Scanning Electron Microscopy

Scanning electron microscopy (SEM) images were acquired using a Zeiss Sigma VP field emission scanning electron microscope equipped with an in-lens electron detector working in the high vacuum mode and an EHT voltage of 5 kV.

#### Analytical Centrifuge

A LUMiSizer Dispersion Analyzer (LUM GmbH, Germany) was used to measure the hydrodynamic diameter of the nanoparticles. LUM 2 mm, PC, Rect. Synthetic Cells (110-132xx) were used as the cuvette, and the density of the nanomaterial is set at 19.32 g/cm^3^ for Au.

#### Small Angle X-ray Scattering

Small angle X-ray scattering (SAXS) data were acquired using the Malvern Panalytical equipment constituted of a diffractometer (Empyrean), a SAXS/WAXS chamber (ScatterX78), and a solid-state detector (PIXcel3D). The incoming slit collimated Cu Kα beam was focalized by an elliptically bent, 1D graded, multilayer X-ray mirror; Cu Kβ contamination was less than 0.1%. Data were fitted using a polydisperse noninteracting Schultz distribution of spheres.

### Cell Culture

HDF cells were cultured in DMEM containing phenol red, supplemented with 10% v/v FBS, 1% MEM nonessential amino acid solution, 1% l-sodium pyruvate, and 1% penicillin–streptomycin mixed solution, in a humidified incubator at 37 °C and 5% CO_2_ conditions.

### Live/Dead Assay

Cell viability quantification was performed by a live/death assay to determine the viability of cells based on esterase activity and plasma membrane integrity after exposition to AuNPs (2 × 10^10^ NPs/ml) for 24 and 48 h. As a positive control, cells were treated with Triton-X detergent at a concentration of 0.05 wt % in PBS for 30 min. After exposure, HDF cells were washed with PBS and stained with a diluted solution of calcein-AM and PI in PBS for 30 min. After being rinsed with PBS, cells were left in fresh PBS media for observation and quantification through a fluorescence microscope. Cell counting was performed using a Keyence BZ-X710 fluorescent microscope, acquiring 10 images in random positions for each well and averaging the results. The measurements were carried out in triplicate.

### SERS Imaging

For SERS studies, HDF cells were grown on 35 mm glass-bottom dishes in complete growth medium in an incubator at 37 °C and 5% CO_2_ for 24 h. Subsequently, the cells were treated for 24 h with 2 × 10^10^ NPs/ml AuNPs diluted in supplemented DMEM cell culture medium, as reported in the literature.^28^ Raman spectra and imaging data of living HDF cells were collected in DMEM media in the presence or absence of ammonia, in a time-dependent manner, with a dedicated Raman device (RAMANtouch, Nanophoton Co., Osaka, Japan). The RAMANtouch spectroscope was operated with an excitation source of 785 nm (excitation power density = 1.4 mW/μm^2^), a 300 gr/mm grating, and a 60× immersion objective lens (NA = 1.0). The spectral resolution was 1.2 cm^–1^ (spectral pixel resolution equal to 0.3 cm^–1^/pixel) and was collected in the range from 300 to 2300 cm^–1^. The RAMANtouch spectroscope was operated in the “line-mode”, acquiring up to 400 simultaneous spectra per line and performing maps of around 140 × 20 μm^2^ with a step size of 0.4 μm between each line. The exposure time for each line was 2 s, and the acquisition was averaged two times per line. The resulting acquisition time was around 4–5 min per map. All the experiments were performed in triplicate, on one cell per each replicate. Reference Raman spectra of pure compounds were also collected with a RAMANtouch spectroscope, as follows: saturated water solutions of cysteine, methionine, cysteic acid, and methionine sulfoxide were mixed with AuNPs and ammonia to reach a final concentration of AuNPs and NH_3_ of 100 μg/mL. Raman spectra were then acquired from the liquid solution, depositing a drop of the sample on an aluminum substrate.

Raman data were processed by averaging more than 4000 spectra per map through RAMAN Viewer software (Nanophoton Co., Osaka, Japan). To the obtained average spectrum, background subtraction, smoothing (Savitsky–Golay smoothing; degree 2, size 9, and height 11), and baseline correction (manually selecting the points representative of the background) were performed by means of LabSpec software (HORIBA, Japan). Subsequently, standard normal variate normalization was performed using SpectraGryph software. Finally, an average of the resulting Raman spectrum for each cell was performed.

### Fluorescence Microscopy for ROS Visualization

HDF cells were grown in complete growth medium in an incubator at 37 °C and 5% CO_2_ for 48 h. Subsequently, cells were treated with NH_3_ (100 μg/mL) for 90 min, keeping them in the incubator chamber. Complete growth medium and tBHP (100 μM) were used as the negative and positive controls, respectively. After, cells were washed twice with PBS and then stained with DCFH-DA solution diluted in culture media, keeping them in the dark inside the incubator chamber. Prior to fluorescence microscopy, cells were washed two times with PBS and then kept in PBS during the analysis. The measurements were carried out in triplicate.

## Results and Discussion

**Figure 1:**
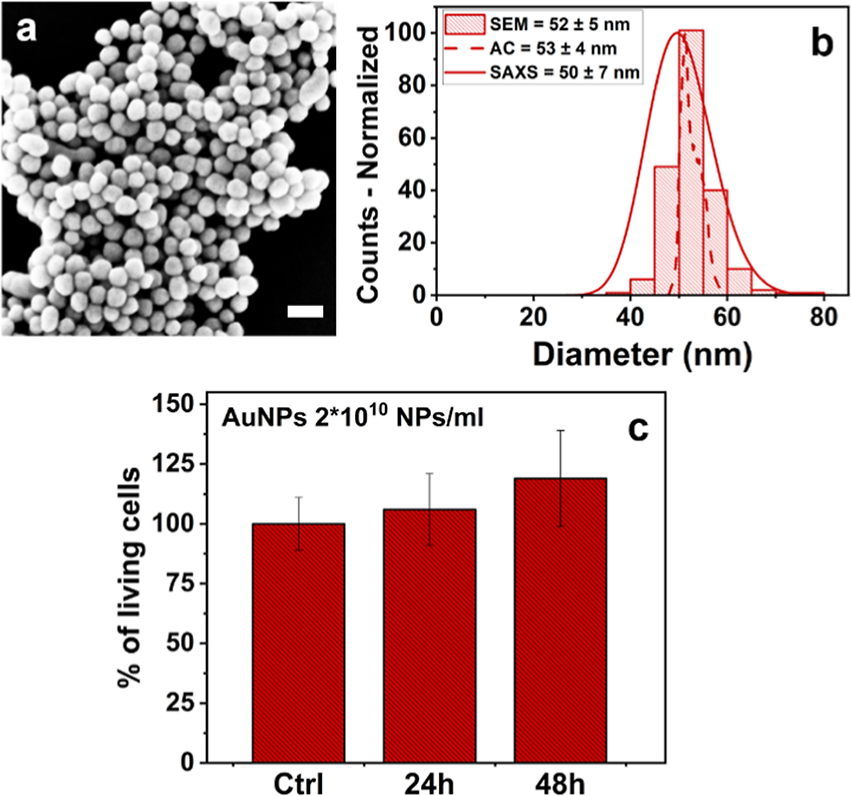
(a) SEM image of AuNPs (scale bar 100 nm); (b) SEM, AC, and SAXS diameter distributions; and (c) quantification of living cells after incubation with AuNPs at 2 × 10^10^ NPs/ml for 24 and 48 h.

AuNPs of size 50 nm are prepared following a modified procedure reported by Bastus et al.^35^ AuNPs seeds are initially synthesized through HAuCl_4_ reduction in the presence of sodium citrate (Au/sodium citrate moles ratio = 2). The obtained nanoparticles had a spherical shape with a mean diameter of 29 ± 4 nm (Figure S1). Starting from these seeds, two subsequent growth steps are performed, which lead to the formation of AuNPs of around 50 nm size (Figure 1a). The SEM image shows the spherical shape of the nanoparticles with a narrow size distribution of 52 ± 5 nm. Analytical centrifugation and SAXS analysis additionally confirmed the average diameter and the small size distribution (Figure 1b).

This size of nanoparticles was chosen for its optimal balance between the strong plasmonic field and low cytotoxicity, as usually reported in the literature.^28,30^ Moreover, no additional functionalization was performed on the AuNPs surface to avoid attenuation of the SERS signal and allow the particles to be distributed only within the cytoplasm without reaching the cell nucleus. To confirm the biocompatibility of the nanoparticles for short time exposures, AuNPs were tested against HDF cells for 24 and 48 h at the concentration used for SERS experiments. Quantification of living cells was performed by the live/dead assay, after staining with calcein-AM and PI diluted solution (Figure S2). This was done as a replacement for typical living cell quantification methods (e.g., WST-8 or other absorbance-based methods), to avoid possible misinterpretation of the results due to the intrinsic absorbance of AuNPs (Figure S1).^37,38^ The exposure to AuNPs resulted in no cytotoxicity against HDF cells for both tested times, as shown in Figure 1c.

**Figure 2:**
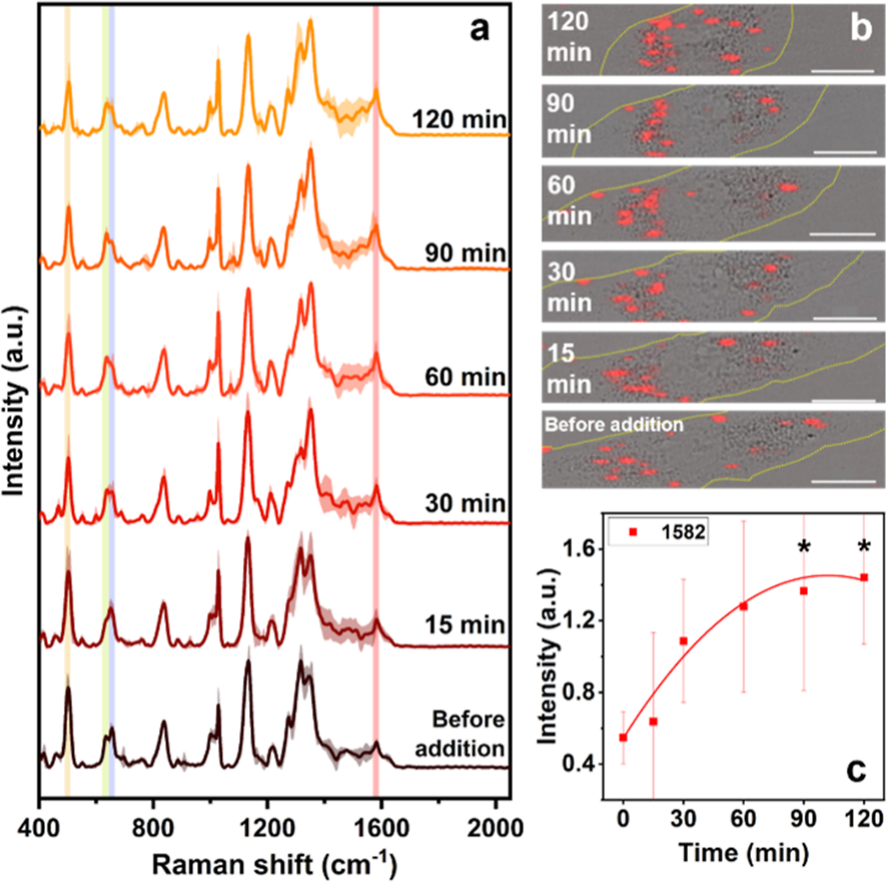
(a) Time-dependent SERS spectra collected from HDF cells being exposed to NH_3_ (100 μg/mL) and (b) SERS maps of AuNP distribution inside a cell, acquired at the respective time intervals (scale bar 20 μm). Yellow lines depict the cell outer membrane and red spots represent the location of AuNP aggregates inside vesicles from which SERS signals derive. (c) Intensity of vibration corresponding to the 1582 cm^–1^ band collected as a function of time when the HDF cells were exposed to NH_3_ (100 μg/mL). *Significant differences from the “0 min” (*P* < 0.05).

SERS experiments were conducted on HDF cells grown onto glass-bottom dishes for 24 h and then treated with AuNPs at 2 × 10^10^ NPs/ml for additional 24 h.^28^ Before ammonia exposure, SERS spectra of the cell were acquired to confirm its initial unstressed state (Figure 2). Subsequently, NH_3_ was added to the culture media to reach a concentration of 100 μg/mL, and SERS images were collected from a single cell in a time-dependent manner. During all the experiments, the culture media were maintained at 37 °C.

From Raman maps acquired before ammonia addition, the accumulation of AuNPs is clearly visible only in the vesicles of the cytoplasmic space (Figure 2b). This confirms the fact that AuNPs of size 50 nm possess the correct dimension to be easily internalized within the cell but without entering the nucleus and potentially causing genotoxicity.^30,39,40^ Following the addition of NH_3_, shrinkage of the cell began to occur, as observed from Figure 2b. Indeed, the vesicles containing AuNPs started to gather and move close to the nucleus, in concomitance with the cell shrinkage process. After 2 h, the cell showed a more spherical shape compared to the initial elongated condition, as commonly associated with the early stages of apoptosis.^41^

The Raman spectra reported in Figure 2a are obtained by averaging the SERS signals from the acquired maps of single cells in triplicate tests. For these spectra, only the Raman bands that demonstrated a noticeable intensity and consistent alterations were taken into consideration during the analysis due to the high complexity of intracellular signals. The Raman bands can be associated with different vibrations of specific biological molecules present inside the cell. A tentative assignment of these signals can be found in Table 1.

**Table 1:** Tentative Assignment to SERS Bands

wavenumber (cm^–1^)	biological molecule	tentative assignment of bond vibration	refs
490–510	proteins	–S–S– bond	(42)–^45^
620–665	proteins	–C–S– bond	(42)–^45^
810–860	proteins and lipids	tyrosine, tryptophan, and C_4_N^+^ and O–C–C–N bonds	(46) and (47)
995–1005	proteins	phenylalanine	(42), (48), and (49)
1020–1035	proteins	phenylalanine (in collagen)	(42), (48), and (49)
1100–1150	proteins and lipids	C–N of proteins and the cis and trans conformations of lipids	(50) and (51)
1200–1235	proteins	amide III (β-sheet)	(50) and (51)
1265–1285	proteins	amide III (α-sheet)	(50) and (51)
1290–1365	proteins and lipids	–CH, –CH_2_, and –CH_3_ in proteins and lipids	(52) and (53)
1582	proteins	phenylalanine/tryptophan	(42), (49), (54), and (55)

The band at ∼502 cm^–1^ can be related to the vibration of the disulfide bond (S–S), while the broad signal between 620 and 660 cm^–1^ can be assigned to the C–S bond, both caused by the sulfur-containing molecules present in proteins.^42−45^ The band centered at ∼835 cm^–1^ is caused by the vibration of tyrosine and tryptophan in proteins and assigned to C_4_N^+^ and O–C–C–N stretching in lipids.^46,47^ The peaks centered at 1000 and 1030 cm^–1^ are assigned to ring breathing vibrations of phenylalanine (Phe) in proteins.^42,48^ The higher intensity of the band at 1030 cm^–1^ can be caused by the large presence of collagen in HDF cells. Indeed, fibroblasts are known as structural cells responsible for producing and secreting a larger amount of extracellular matrix components, such as collagen proteins, than other human cells.^56,57^ This can result in an enhanced signal derived from Phe residues present in collagen.^49^

The intense band between 1100 and 1150 cm^–1^ is related to the C–N vibration of proteins and the cis and trans conformations of lipids,^50,51^ while the signals centered at ∼1215 and ∼1270 cm^–1^ are caused by amide III vibrations in β-sheet and α-helix structures of proteins, respectively.^50,51^ The large band between 1290 and 1365 cm^–1^ can be attributed to all the vibrations of alkyl groups in proteins and lipids.^52,53^

Eventually, the peak at ∼1582 cm^–1^ is assigned to phenylalanine and tryptophan vibrations.^42,49,54,55^ This latter band is reported to increase in intensity during protein unfolding and apoptosis processes.^43,47,48^ After 15 min from ammonia addition, this band showed an increasing trend over time (Figure 1c). This phenomenon can be attributed to the denaturation of proteins,^58^ caused in turn by the presence of ROS and RNS generated after NH_3_ exposure.^7,59^ Indeed, the continuous stressful condition to which the cells are exposed enables protein denaturation to occur, allowing the internal hydrophobic Phe and Trp residues to interact with AuNPs, thus enhancing the intensity of the peak at ∼1582 cm^–1^.

**Figure 3:**
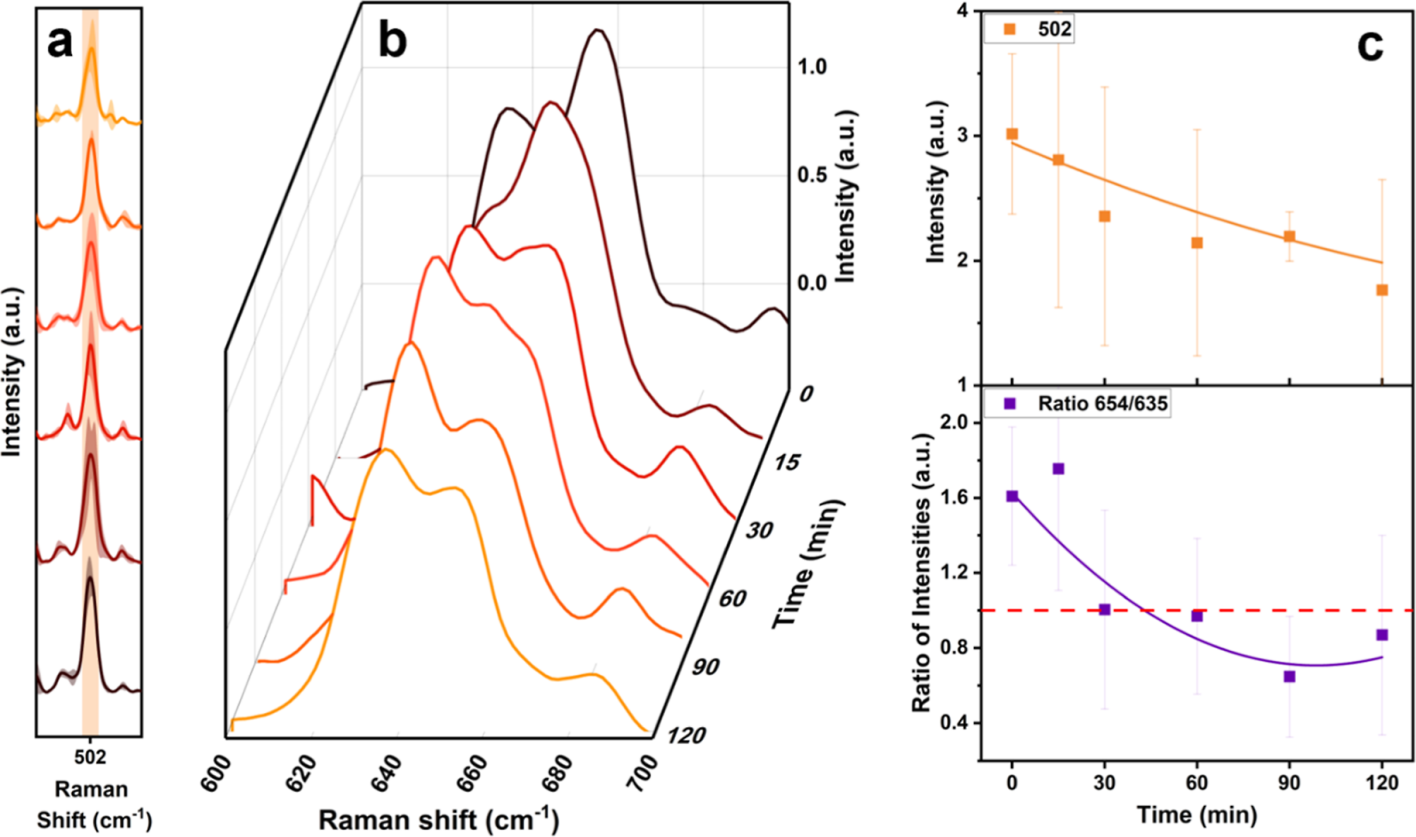
(a) Magnification of the Raman bands at 502 cm^–1^ (S–S bond) and (b) at 620–665 cm^–1^ regions (C–S bonds) and (c) intensity of the vibration corresponding to the 502 cm^–1^ band, and intensity ratio between 654 and 635 cm^–1^ bands collected as a function of time when the HDF cells were exposed to NH_3_ (100 μg/mL).

A confirmation to this process comes from the changes in the signal at 502 cm^–1^ related to the S–S bond and the signal between 620 and 665 cm^–1^ related to the C–S vibration (Figure 3). After ammonia addition, the band at 502 cm^–1^ shows a decreased intensity, which is accompanied by a variation in the shape of the 620–665 cm^–1^ peaks. This latter band is given by the combination of two different signals: (i) the one at 635 cm^–1^ caused by the vibrations of the C–S bond in oxidized cysteine (cysteic acid, C–SO_3_H) and oxidized methionine (methionine sulfoxide, C–SO–CH_3_) and (ii) the one at 654 cm^–1^ assigned to the C–S bond in the reduced form (C–SH and C–S–CH_3_).^43−45^ Cysteic acid and methionine sulfoxide are the corresponding oxidized amino acids of cysteine and methionine, respectively, which are generally formed during the protein oxidation phenomenon in stressed cells.^60−62^ After 30 min, the relative intensity of the 654 cm^–1^ band decreases with respect to the one at 635 cm^–1^ (Figure 3b,c), thus resulting in an increase in the band related to C–SO_3_H/C–SO–CH_3_ bonds and a decrease in the one assigned to C–SH/C–S–CH_3_ bonds (Figure 3). This can be ascribed to the continuous presence of an oxidative stress condition inside the cell, which causes the breaking of disulfide bonds and the oxidation of cysteine and methionine residues, thus leading to protein denaturation.^43,46,48,58,63^

Over time, a continuous decrease in the 502 cm^–1^ band is observed; however, due to the large variance between the averaged spectra, no statistical differences can be attributed between the initial and final conditions. Attention must therefore be paid to this band, especially in the initial stages of mild cellular stress treatments. In fact, concomitant antioxidative processes (e.g., the oxidation of glutathione) could cause discrepancies in the final interpretation of the intensity of the S–S bond band. A hypothesis on the oxidative process can however still be made since a complete inversion in the intensities of the 635–654 cm^–1^ coupled bands is observed. The cysteine residues released by the S–S bond break undergo oxidation caused by the ROS/RNS present in stressed cells. This phenomenon also causes the oxidation of the methionine residues, which, by converting into methionine sulfoxide, lead to an additional increase in the 635 cm^–1^ peak intensity.

**Figure 4:**
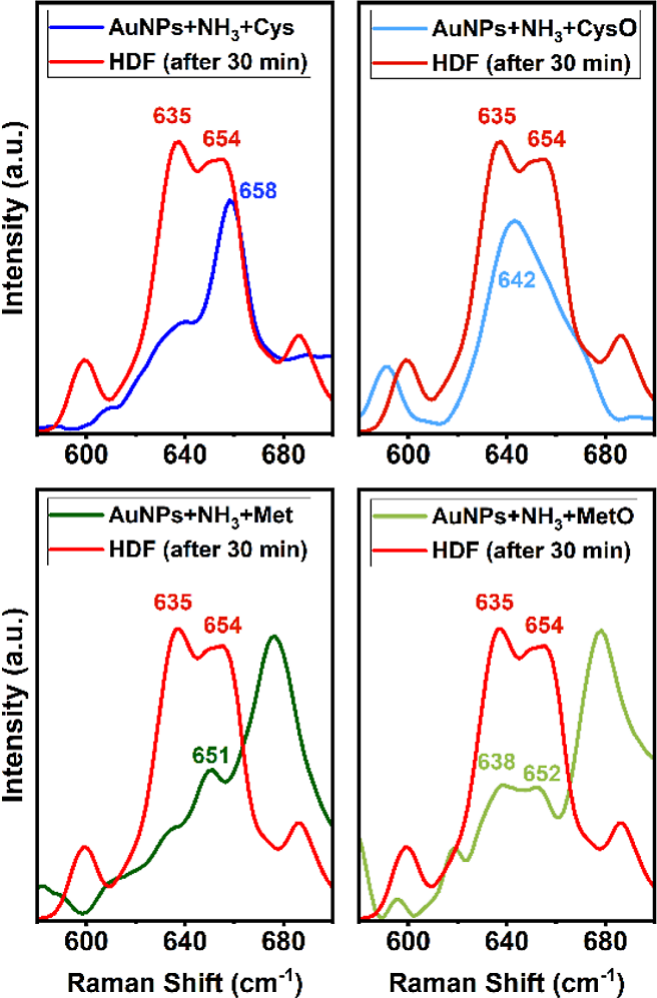
SERS spectrum of a selected region representative of the HDF cell (exposed to NH_3_ for 30 min) compared to the corresponding region of the SERS spectrum for cysteine (Cys), cysteic acid (CysO), methionine (Met), and methionine sulfoxide (MetO).

To further verify the contribution to the bands observed between 620 and 665 cm^–1^, we acquired the Raman spectra under 785 nm excitation of pure cysteine (Cys), cysteic acid (CysO), methionine (Met), and methionine sulfoxide (MetO) in the presence of AuNPs and NH_3_. The C–S vibration region of the Raman spectrum collected from the HDF cells exposed to NH_3_ for 30 min and the spectrum of the amino acids is shown in Figure 4. The spectral characteristics found in the HDF spectrum after 30 min show strong similarities to the corresponding bands of the amino acids. Even though the spectral peaks present minor shifts and slight differences in relative intensities, the Raman bands at 642 cm^–1^ for CysO and 638 cm^–1^ for MetO can be related to the band at 635 cm^–1^ in ammonia-treated HDF cells. On the contrary, Cys and Met residues do not present any signal around 635 cm^–1^ but show only peaks at 658 and 651 cm^–1^, respectively. These results correctly support the hypothesis that the 635 cm^–1^ peak present in treated HDF cells is caused by the sum of the C–S vibrations in cysteic acid and methionine sulfoxide, while the one at 654 cm^–1^ is due to the C–S vibration in the reduced form (C–SH and C–S–CH_3_).

To confirm that these oxidative processes are mainly caused by ammonia exposure, SERS experiments have been performed on untreated cells (Figures S3 and S4). Results from Figure S3 show a slight cell shrinkage and a small increase in the 1582 cm^–1^ Raman band on the HDF cell, though with a lower degree than that of the one shown during NH_3_ exposure. Moreover, no decrease in the S–S bond signal at 502 cm^–1^ is observed, and the switching of the intensities of 635 and 654 cm^–1^ never occurred after 2 h of observation, thus proving the absence of high oxidative stress conditions. The slight variation observed in control cells can be attributed to the increase in the pH of the culture media over time, caused by the lack of CO_2_ exposure during the measurements (Table S1). CO_2_ is an important component useful to maintain the optimal environment for cell growth and regulate the pH of the culture media. A chamber environment of 5–10% CO_2_ is required for the most widely used buffering method and results in a constant pH of 7.2–7.4.^64^ Therefore, the absence of CO_2_ incubation during SERS experiments resulted in a slow increase of the pH over time, leading to small variations in the cell’s metabolism.

**Figure 5:**
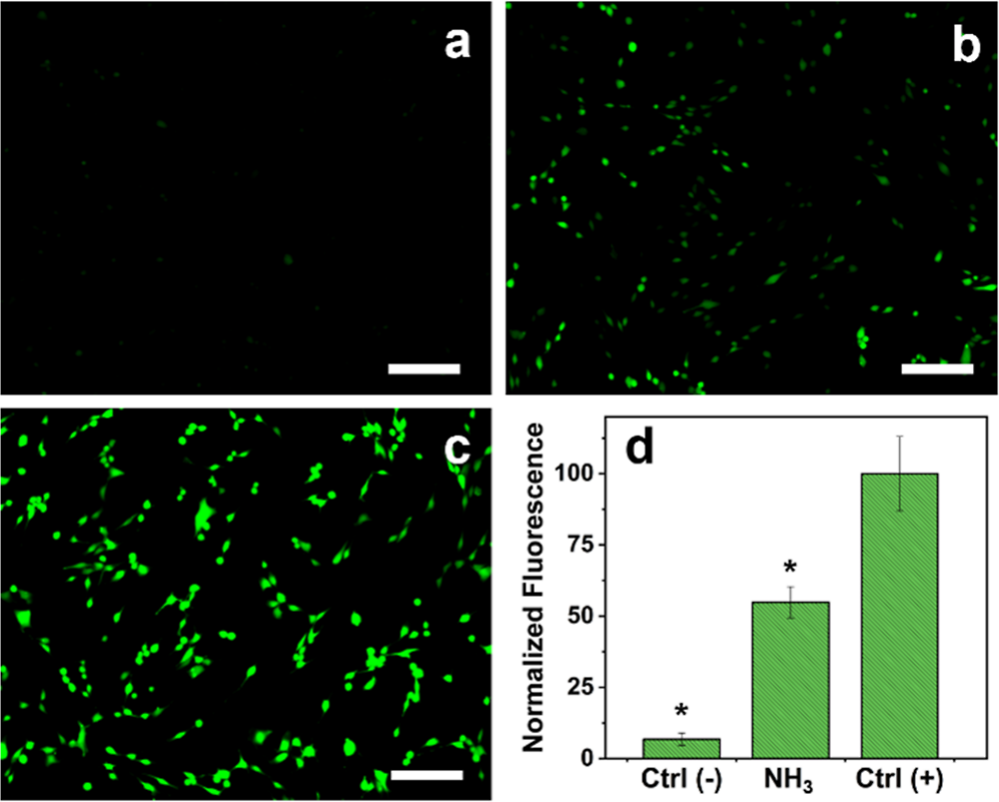
Fluorescence microscopy of HDF cells (a) untreated (negative control), (b) exposed to NH_3_ (100 μg/mL) for 90 min, and (c) treated with tBHP (100 μM) (scale bar 200 μm) and (d) normalized fluorescence intensity profile with respect to tBHP (positive control). *Significant differences from the positive control (*P* < 0.05).

In order to confirm the formation of ROS and RNS, fluorescence microscopy has been performed on HDF cells exposed to 100 μg/mL ammonia for 90 min, using DCFH-DA as a fluorescent reporter molecule (Figure 5). As expected, untreated cells show the quasi-absence of the fluorescence signal, proving their unstressed condition. On the contrary, the presence of NH_3_ at 100 μg/mL and tBHP (positive control) led to the formation of ROS and RNS, which in turn oxidate DCFH molecules into a green fluorescent DFC compound. In Figure 5, it is possible to observe a different intensity between the signals generated from NH_3_ and tBHP. tBHP is a well-known pro-oxidant compound, which causes a high production of ROS molecules due to the presence of the hydroperoxide group (–OOH), which is easily cleaved and leads to the release of reactive radicals (^•^OH). On the contrary, ammonia shows a lower formation of ROS and RNS as its mechanism of action leads to a more general imbalance of the cellular machinery. The slight presence of ROS formation in the negative control is most likely due to the sample handling processes and the maintenance of the cells in PBS during the measurement, which, not being the complete growth medium, causes a stress effect. These results proved that the exposure to ammonia led to an increase in the oxidized condition inside the cells, which is the cause of the subsequent oxidation and denaturation of the proteins.

Ammonia is a well-known toxic compound that is reported to cause the formation of ROS and RNS through an oxidative stress process.^6−8^ Oxidative stress conditions occur when there is an imbalance between the production of ROS/RNS and the ability of the cell to detoxify them or repair the damage that they cause. Ammonia can enter cells through passive diffusion or active transport mechanisms. Inside the cell, NH_3_ can be metabolized in the mitochondria as part of the urea cycle and converted it into less toxic urea. If the ammonia concentration in too high, mitochondrial dysfunction can occur and lead to a decrease in ATP synthesis and an increased formation of free radicals.^59^ Indeed, according to the literature, when cells are exposed to pathophysiological levels of ammonia, it can cause a significant rise in the mitochondrial NAD^+^/NADH ratio, leading to excessive production of ROS.^65^ Overproduction of ROS can damage the respiratory chain and increase mitochondrial permeability, thus affecting the electron transport chain. Mitochondria have been suggested to be the center of the apoptotic pathways, and therefore, impairing of their regular function can lead to the activation of the apoptosis processes.^66^ Studying the ROS and RNS effects inside the cell is therefore crucial to deeply understand the metabolic reactions that toxic molecules can cause to the organisms. The SERS technique is a valuable option to investigate these processes, as reported in the literature.^47,50^ The observation of specific signals caused by different toxicants or by detrimental conditions could shed light on the hidden and elusive metabolic mechanisms, especially in those cases in which specific Raman active bonds are formed.

## Conclusions

In our work, we exploited the potential of the surface enhanced Raman phenomenon produced by AuNPs to investigate the effects of ammonia at the single-cell level. During NH_3_ exposure, ROS and RNS formation occurred, as revealed by fluorescence microscopy, leading to an oxidative condition inside the cell. This stressed condition caused the oxidation of the cysteine and methionine amino acids into cysteic acid and methionine sulfoxide and successively led to the denaturation of the cell’s proteins. Moreover, after exposure to the toxic compound, we observed an increase in the apoptosis signal derived from phenylalanine and tryptophan together with the occurrence of the cell shrinkage phenomenon. These outcomes provided a further understanding of the mechanism of ammonia toxicity at the single-cell level, additionally proving the great potential of the SERS technique to detect early and fine signs of metabolic changes.

## Supporting Information Available

Additional NP characterizations, fluorescence images of HDF cells stained with calcein-AM and PI, time-dependent SERS spectra and images of the untreated control cell, and pH values of culture media over time (PDF)

## Abbreviations

HDF: human dermal fibroblast
SERS: surface enhanced Raman spectroscopy
AuNPs: gold nanoparticles
ROS: reactive oxygen species
RNS: reactive nitrogen species
Cys: cysteine
CysO: cysteic acid
Met: methionine
MetO: methionine sulfoxide
