# Supplementary material for: Ammonia Toxicity and Associated Protein Oxidation: A Single-Cell Surface Enhanced Raman Spectroscopy Study
Source: Chem Res Toxicol. 2023 Dec 26;37(1):117–25. doi: 10.1021/acs.chemrestox.3c00368 (PMC10792663; doi:10.1021/acs.chemrestox.3c00368)
Supplement: Supplementary file 1 — tx3c00368_si_001.pdf [file tx3c00368_si_001.pdf]

SUPPORTING INFORMATION

Ammonia toxicity and Associated Protein oxidation: A Single-cell Surface Enhanced Raman Spectroscopy study

Davide Redolfi-Bristol,[*1,3,7] *Alessandro Mangiameli,*[1,7] *Kenta Yamamoto,*[3] *Elia Marin,*[1] *Wenliang Zhu,*[1] *Osam Mazda,*[3] *Pietro Riello,*[7] *Giuseppe Pezzotti,*[*1,2,3,4,5,6,7]

[1] Ceramic Physics Laboratory, Kyoto Institute of Technology, Sakyo-ku, Matsugasaki, 606-8585, Kyoto, Japan

[2] Department of Molecular Genetics, Institute of Biomedical Science, Kansai Medical University, 2-5-1 Shinmachi, Hirakata, Osaka 573-1010, Japan

[3] Department of Immunology, Kyoto Prefectural University of Medicine, Kamigyo-ku, Kyoto, 602-8566, Japan

[4] Department of Dental Medicine, Graduate School of Medical Science, Kyoto Prefectural University of Medicine, Kamigyo-ku, Kyoto 602-8566, Japan

[5] Department of Orthopedic Surgery, Tokyo Medical University, 6-7-1 Nishi-Shinjuku, Shinjuku-ku, 160-0023 Tokyo, Japan

[6] Department of Applied Science and Technology, Politecnico di Torino, Corso Duca degli Abruzzi 24, 10129 Torino, Italy

[7] Dipartimento di Scienze Molecolari e Nanosistemi, Università Ca' Foscari di Venezia, Via Torino 155, 30172 Venezia, Italia

TABLE OF CONTENT

FIGURE S1. Additional characterization of AuNPs seeds and of AuNPs of 50 nm……………………………S2

FIGURE S2. Fluorescence images of HDF cells stained with Calcein-AM and Propidium Iodide………………..S2
FIGURE S3. Time-dependent SERS spectra and images of untreated control cell……………………………….S3
FIGURE S4. Magnified portions of time-dependent SERS spectra of untreated control cell…………………….S4
TABLE S1. pH values of culture media during time…………………………………………………………….S3

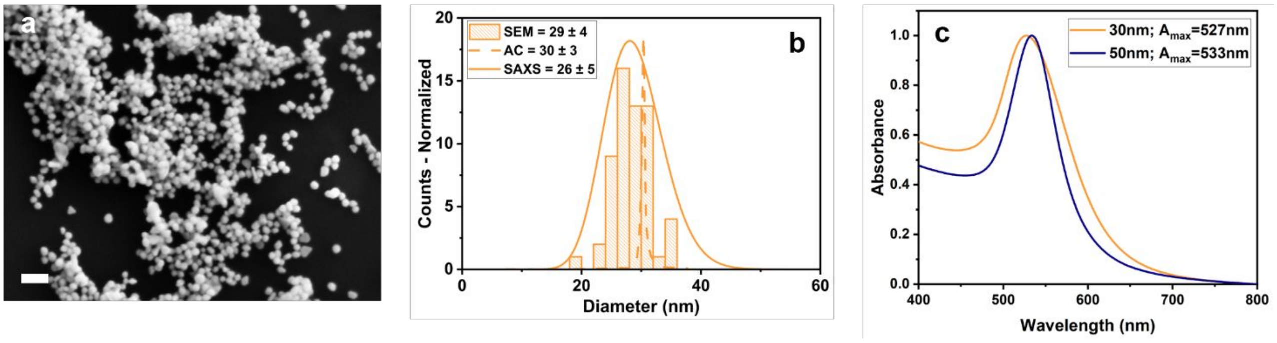

Figure S1. (a) SEM Image of AuNPs seed (scale bar 100nm) and (b) SEM, AC and SAXS diameter distribution of AuNPs seed. (c) UV-Visible absorption spectra of AuNPs seed and AuNPs of 50nm

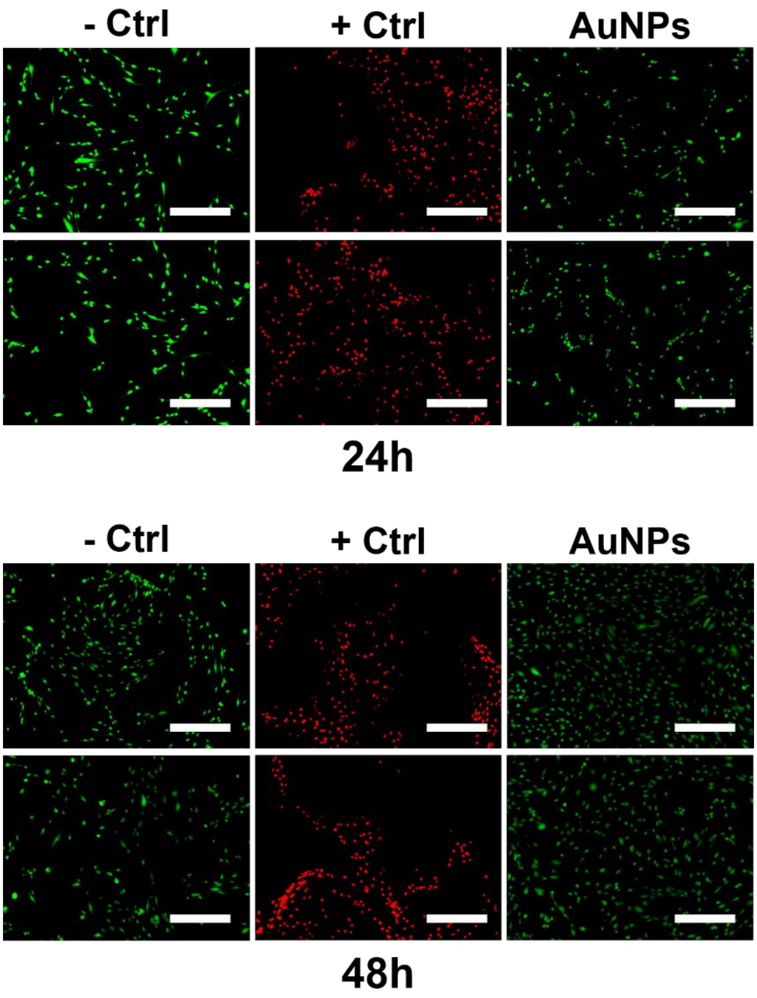

Figure S2. Representative images of Calcein-AM and Propidium Iodide (PI) stained HDF cells exposed to AuNPs for 24h and 48h and to Triton-X for 30 min as positive control (scale bar: 200 μm)

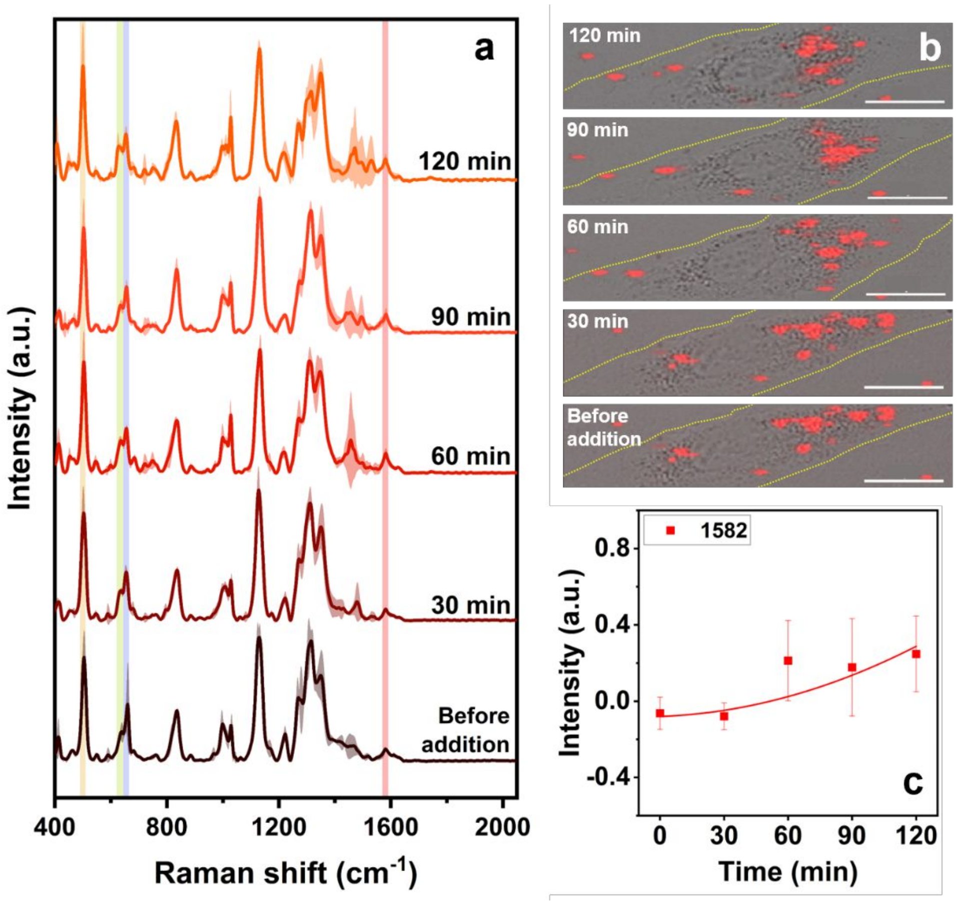

Figure S3. (a) Time dependent SERS spectra collected from untreated HDF cells and (b) SERS maps of AuNPs distribution inside the cell, acquired at the respective time intervals (scale bar 20 μm). Yellow lines depict the cell outer membrane; red spots represent the location of AuNPs aggregates inside vesicles from which SERS signals derive (c) Intensity of vibration corresponding to 1582 cm^{-1} band collected as a function of time

Table S1. pH values of Culture Media during time

Time (min)	Culture Media (CM)	CM+NH_3 100 μg/ml
0	7.76 ± 0.01	8.62 ± 0.04
30	7.86 ± 0.05	8.62 ± 0.01

60	7.88 ± 0.03	8.62 ± 0.01
90	7.98 ± 0.03	8.66 ± 0.01
120	8.04 ± 0.03	8.65 ± 0.01

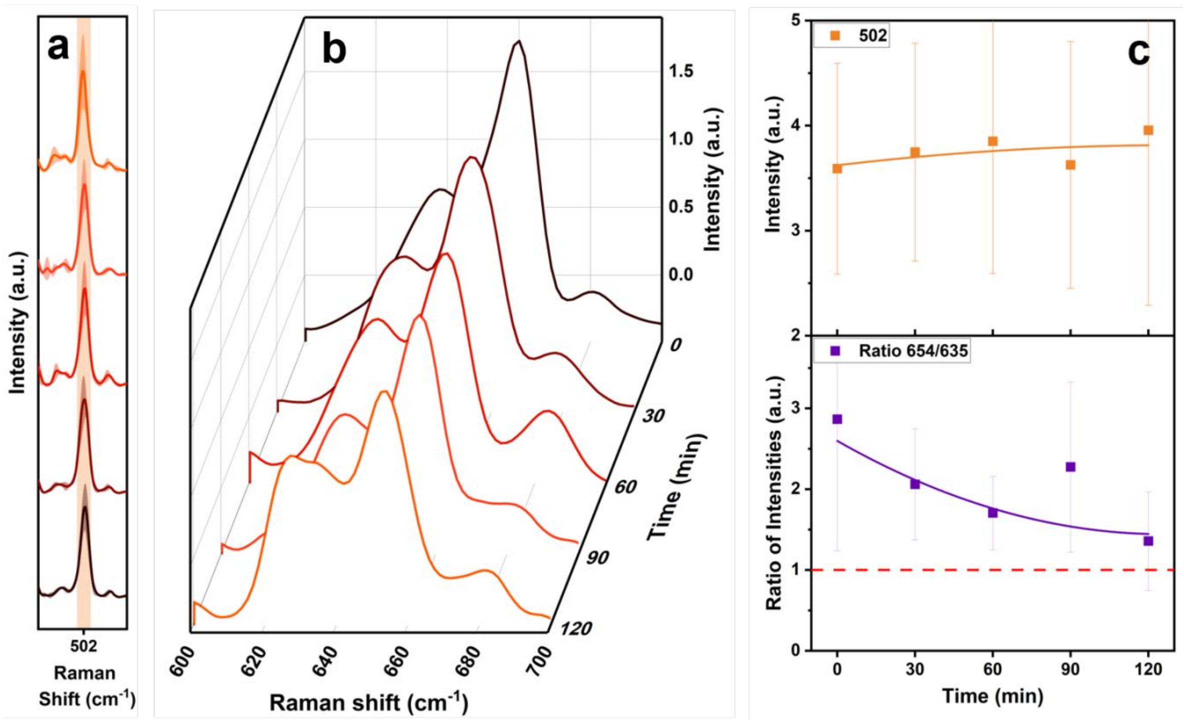

Figure S4. (a) Magnification of the Raman bands at 502 cm^{-1} (S-S bond) and (b) at 620-665 cm^{-1} region (C-S vibration); (c) intensity of the vibration corresponding to 502 cm^{-1} band, and intensity ratio between 654 and 635 cm^{-1} bands collected as a function of time for untreated cells.
